# SARS-CoV-2 Main Protease Targets Host Selenoproteins and Glutathione Biosynthesis for Knockdown via Proteolysis, Potentially Disrupting the Thioredoxin and Glutaredoxin Redox Cycles

**DOI:** 10.3390/antiox12030559

**Published:** 2023-02-23

**Authors:** Ignacio A. Gallardo, Daniel A. Todd, Stella T. Lima, Jonathan R. Chekan, Norman H. Chiu, Ethan Will Taylor

**Affiliations:** Department of Chemistry and Biochemistry, University of North Carolina Greensboro, Greensboro, NC 27403, USA

**Keywords:** COVID-19, DNA synthesis, glutathione, RNA, SARS-CoV-2, selenium, SELENOF, SELENOP, selenoprotein, thioredoxin reductase 1

## Abstract

Associations between dietary selenium status and the clinical outcome of many viral infections, including SARS-CoV-2, are well established. Multiple independent studies have documented a significant inverse correlation between selenium status and the incidence and mortality of COVID-19. At the molecular level, SARS-CoV-2 infection has been shown to decrease the expression of certain selenoproteins, both in vitro and in COVID-19 patients. Using computational methods, our group previously identified a set of six host proteins that contain potential SARS-CoV-2 main protease (M^pro^) cleavage sites. Here we show experimentally that M^pro^ can cleave four of the six predicted target sites, including those from three selenoproteins: thioredoxin reductase 1 (TXNRD1), selenoprotein F, and selenoprotein P, as well as the rate-limiting enzyme in glutathione synthesis, glutamate-cysteine ligase catalytic subunit (GCLC). Cleavage was assessed by incubating recombinant SARS-CoV-2 M^pro^ with synthetic peptides spanning the proposed cleavage sites, and analyzing the products via UPLC-MS. Furthermore, upon incubation of a recombinant Sec498Ser mutant of the full TXNRD1 protein with SARS-CoV-2 M^pro^, the predicted cleavage was observed, destroying the TXNRD1 C-terminal redox center. Mechanistically, proteolytic knockdown of both TXNRD1 and GCLC is consistent with a viral strategy to inhibit DNA synthesis, conserving the pool of ribonucleotides for increased virion production. Viral infectivity could also be enhanced by GCLC knockdown, given the ability of glutathione to disrupt the structure of the viral spike protein via disulfide bond reduction. These findings shed new light on the importance of dietary factors like selenium and glutathione in COVID-19 prevention and treatment.

## 1. Introduction

The world has been in crisis since early in 2020 over the Coronavirus Disease-2019 (COVID-19) pandemic, brought on by the emergence of the Severe Acute Respiratory Syndrome Coronavirus Virus 2 (SARS-CoV-2). While first identified in Wuhan, China in December of 2019, the WHO did not officially deem COVID-19 a pandemic until 11 March 2020, as cases began to spread globally at an alarming pace [1].

The risk of continuing emergence of new SARS-CoV-2 variants that can evade our current vaccines and therapeutic monoclonal antibodies [2], along with increasing numbers of “Long COVID” cases that arise consequent to the ever-expanding cumulative number of COVID-19 survivors [3], highlight a need for complementary therapeutic modalities that can moderate viral pathogenicity via effects on host factors, which are genetically stable compared to viral targets. Dietary factors including vitamins, minerals, and regulatory molecules like glutathione (GSH) are potentially well suited to this purpose, although their mechanisms, efficacy, and potential utility in the treatment of COVID-19 remain controversial, e.g., in the case of vitamin D, which continues to elicit diametrically opposed claims documented in numerous apparently well conducted studies [4,5,6,7,8].

One of the first micronutrients for which evidence of a significant role in COVID-19 emerged, even within the first months of the pandemic, is the trace element selenium (Se) [9,10,11]. The fact that multiple research groups from different countries studied this question intensely, almost from the outset of the pandemic, is not that surprising in the light of over 40 years of accumulating evidence that has firmly demonstrated links between Se status and the clinical outcome of various viral infections, including HIV-1, coxsackieviruses, hepatitis viruses, hantaviruses, influenza virus, and most recently, SARS-CoV-2, as detailed in many recent reviews [12,13,14,15,16,17,18,19]. The sheer number of new review articles on the role of Se in viral infections, and its significance for COVID-19, reflects the greatest rekindling of interest in this topic in several decades.

In all of the reviews cited above, the fundamental roles of the known selenoproteins in various biological processes such as immunity and inflammation has typically been the primary focus; these known mechanisms are then used to explain how Se deficiency in populations may exacerbate viral infections and COVID-19 in particular. In their reviews, Rayman and coworkers have gone somewhat deeper, also considering the potential importance of (1) direct antiviral roles of low molecular weight redox-active Se species, and (2) the possibility that some viruses, and SARS-CoV-2 specifically, employ mechanisms designed to obstruct critical selenoprotein mediated defense mechanisms via active suppression of selenoprotein gene expression, potentially at both the RNA and protein levels [14,19].

The importance of Se for the immune response was already emerging in the late 1970s [20], and by the 1990s, it was well understood to be an essential nutrient for immune function, as well as for antioxidant defenses and cellular survival [21,22,23]. This decades-old foundational understanding has now been greatly expanded by the discovery of additional human selenoproteins and their roles in the immune system [24,25]. *This knowledge has tended to favor a mindset that sub-par expression of specific selenoproteins leading to impaired immunity is the major basis of links between Se and viral pathology, which hence are fundamentally a problem limited to Se deficient populations*, and possibly those with comorbidities that might impair selenoprotein function. This model as applied to SARS-CoV-2 is typified by Figure 2 in the review by Bermano et al. [12]. However, *if it is true that inadequate levels of critical selenoproteins undermine immunity and other essential host processes*, thereby enhancing vulnerability to viral pathogenesis, then *it should be equally true that a virus could benefit by actively suppressing the levels of those same selenoproteins*. If so, individuals with adequate dietary intake but high viral loads might be suffering from impaired selenoprotein function due to viral knockdown, and thus potentially might benefit from increased supra-nutritional intake of Se.

Based on further analysis of the data from Zhang et al., correlating Se status (based upon hair analysis) with COVID-19 recovery rates in Chinese cities [9], Figure 1 of Rayman et al. [19] suggests that, indeed, there *is* a protective effect of Se dietary intakes at levels that exceed those previously shown to be sufficient to optimize the expression of critical selenoproteins like glutathione peroxidase 1 (GPX1) and selenoprotein P (SelenoP). This implies that there is more going on with Se in COVID-19 than simply the rectification of a dietary deficiency.

Furthermore, in an analysis of the effects of SARS infection on expression of selenoprotein mRNAs in Vero cells, Wang et al. reported that six of the 25 known selenoproteins mRNAs were significantly suppressed in infected cells [26]. In the case of thioredoxin reductase 3 (TXNRD3), evidence was also presented consistent with the possibility of an antisense interaction between the selenoprotein mRNA and the viral mRNA as a possible mechanism of mRNA suppression. These results strongly support the hypothesis that SARS-CoV-2 is actively engaged in the mRNA suppression-based knockdown of a number of host selenoproteins, including glutathione peroxidase 4 (GPX4), TXNRD3, and four endoplasmic reticulum (ER)-resident selenoproteins [26].

Evidence for a possible active selenoprotein knockdown strategy by SARS-CoV-2 *at the protein level* was presented by Taylor and Radding [27]. Using web server-based computational methods, we identified short protein sequences in a handful of host proteins that closely match the known target sequences of the SARS-CoV-2 main protease (M^pro^, also called the 3CL protease). Candidate M^pro^ cleavage sites were identified in four selenoproteins: selenoprotein F (SelenoF), SelenoP, GPX1, and thioredoxin reductase 1 (TXNRD1), as well as in two conventional proteins: glutaredoxin (GLRX-1) and the catalytic subunit of γ-glutamate cysteine ligase (GCLC), the rate-limiting enzyme for GSH synthesis. Further examination using available 3D structures suggested that all of these potential M^pro^ cleavage sites are close to the protein surface where they would be accessible to the viral protease, as shown in Figure S3 of reference [27].

It should be noted that other confirmed and unconfirmed cellular targets of SARS-CoV-2 proteases, both M^pro^ and the viral “papain-like protease” (PLpro), have been identified [28,29,30,31]. Experimentally confirmed M^pro^ targets include three proteins involved in host innate immune responses: NLR Family Pyrin Domain Containing 12 (NLRP12), Interleukin-1 Receptor-Associated Kinase 1 (IRAK1), and TGF-Beta Activated Kinase 1 (TAB1), and in addition, C-Terminal-Binding Protein 1 (CTBP1), a protein involved in control of cell development, oncogenesis, and apoptosis [29,30].

The primary function of viral proteases is for virion maturation via viral polyprotein processing, to form the functional structural components of the virion, as well as viral enzymes and regulatory proteins. However, it is also well established that some viral proteases have coevolved to additionally target specific *host* proteins, whose knockdown may facilitate some aspects of viral replication or pathogenesis, e.g., as reviewed by Blanco et al. in the case of HIV-1, as just one example [32]. In the case of GSH biosynthesis and the selenoprotein TXNRD1 as potential viral protease targets [27], there is a clear rationale as to how their knockdown would assist the replication of an RNA virus like SARS-CoV-2. By interfering with the two essential redox cycles needed to sustain the action of ribonucleotide reductase, proteolytic knockdown of both TXNRD1 and GCLC is consistent with a viral strategy to inhibit DNA synthesis, to conserve the pool of ribonucleotides for increased virion production [33]. This hypothesis will be examined in detail in Section 4.

**Figure 1:**
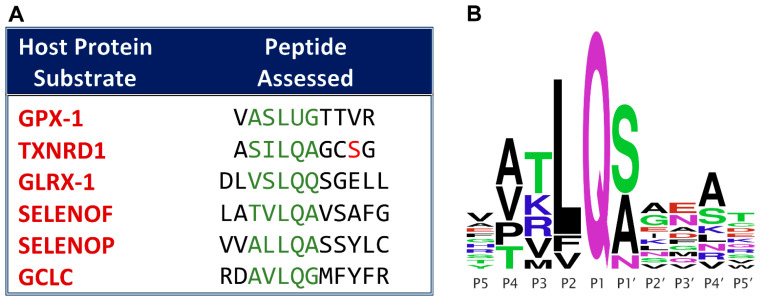
**(A) Synthetic peptides used for M^pro^ cleavage assessment of previously predicted sites from the host proteins listed** [27]. The letters shown in green correspond to the P4 through P1′ positions that are most important for recognition by the enzyme. The red S in TXNRD1 is mutated from a selenocysteine in the wild type protein. **(B) Consensus sequence logo for known SARS-CoV-2 M^pro^ cleavage sites in viral nonstructural proteins**. Cleavage occurs between the conserved glutamine (Q) in the P1 position and the adjacent residue in the P1′ position. The letters stacked vertically in a given position (representing amino acid residues in single letter protein code) show the natural variation tolerated by the protease active site in that position. Simplified from Figure 1 in [27].

**Figure 2:**
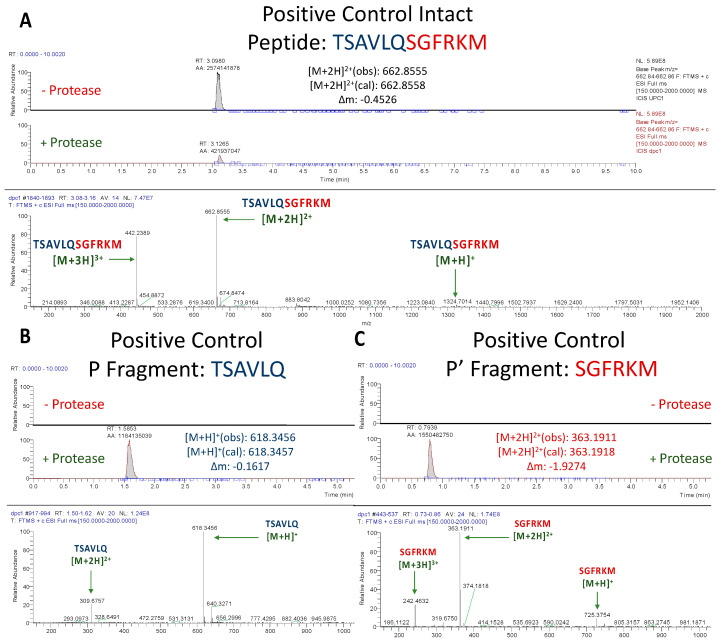
**UPLC-MS Data for Positive Control M^pro^ Substrate.** Depicted are the extracted ion chromatograms for the samples with and without protease (**top**) and the mass spectra (**bottom**) for (**A**) the Intact Peptide, (**B**) the P Fragment, and (**C**) the P′ Fragment. The normalization levels of the base peaks for the P and P′ fragments were 2.39 × 10^8^ and 3.99 × 10^8^, respectively. High resolution mass spectrometric analysis corroborates the identities of each base peak in the corresponding extracted ion chromatogram.

Thus, the principal aim of this work was to experimentally assess the functionality of the potential M^pro^ cleavage sites in the six host proteins identified previously, listed in Figure 2 of Taylor and Radding [27], and in simplified format here in Figure 1. Cleavage was assessed under buffered cell-free conditions, by incubating recombinant SARS-CoV-2 M^pro^ with synthetic peptides spanning the proposed cleavage sites, and analyzing the products via UPLC-MS. Our results showed that the predicted cleavage site candidates in four of the six proteins actually function as M^pro^ substrates. Because the predicted cleavage site in TXNRD1 is right at the protein C-terminus, and is expected to generate a five-residue P′ fragment that includes the essential C-terminal redox center of thioredoxin reductase, we were also able to demonstrate this cleavage from a full 499-residue TXNRD1 protein, under similar cell-free conditions. For this purpose, SARS-CoV-2 M^pro^ was incubated with a recombinant Sec498Ser mutant of the TXNRD1 protein, which generated the same five-residue peptide P′ fragment as the decamer shown for TXNRD1 in Figure 1. The significance of these findings for redox-related selenoprotein-based mechanisms in COVID-19 pathogenesis will be reviewed in Section 4.

## 2. Materials and Methods

### 2.1. Peptide Cleavage Materials

Peptides ranging from 10- to 12-mer were utilized, which incorporated the five or six amino acid residues immediately preceding and following the proposed cleavage sites predicted via the web server tools NetCorona [34], Procleave [35], and PROSPER [36], as described previously [27]. The only exception was that of TXNRD1, whose selenocysteine (U) in the 4th (P4′) position following the cleavage site was replaced by serine. This choice was made for several reasons, and was influenced by the need to compare the cleavage of the isolated peptide with that of the entire TXNRD1 protein (see below). Because of the differences between bacterial and eukaryotic co-translational mechanisms necessary for the incorporation of selenocysteine at UGA codons, recombinant mammalian selenoproteins cannot be produced by cloning and expression of the human mRNAs in bacteria (see Section 2.2). Additionally, the presence of the geminal cysteine residue at TXNRD1 position 497 would have led to the possibility of both reduced and oxidized forms of the Cys-Sec redox center being present, thus confounding the mass spectrometric results. Finally, because the P4′ position is of little importance for M^pro^ recognition [27], substitution at this position is unlikely to have a major impact on cleavage, as illustrated by the extensive substrate variability at this position, shown in Figure 1B. Serine was chosen as a replacement due to the fact that it is isosteric with selenocysteine, and is sometimes inserted at UGA codons under low Se conditions, making it the most natural choice. Hence, this substitution was incorporated both into the model peptide and the mutant TXNRD1 protein studied.

With the exception of GPX1, all of the chosen peptides, including the 12-mer positive control, were synthesized by GenScript (Piscataway, NJ, USA). The 10-mer GPX-1 peptide was synthesized by Thermo Scientific (Waltham, MA, USA). The recombinant SARS-CoV-2 M^pro^ (which was based on NCBI Reference Sequence YP_009725301) and the recommended M^pro^ buffer were obtained from BPS Bioscience (San Diego, CA, USA). The NCBI Reference Sequence accession numbers for the proteins from which the synthesized peptides were derived were as follows: TXNRD1: NP_877393.1; GCLC: NP_001309424.1; SelenoF: NP_004252.2; SelenoP: NP_005401.3; GLRX1: NP_001112362.1; GPX1: NP_000572.2.

### 2.2. Expression and Purification of TXNRD1 Protein

A codon-optimized TXNRD1 gene with the Sec498Ser mutation was synthesized by TwistBioscience (based on NCBI Reference Sequence NP_877393.1). The associated primers were designed by and purchased from IDT, bearing the forward sequence 5′-GGTGCCGCGCGGCAGCCATATGGGATGTGCAGAGGGAAAAGCAGTAGC-3′ and the reverse sequence 5′-GGTGGTGGTGGTGCTCGAGTCATCCGCTACATCCTG-CCTGTAAGATCGAGG-3′.

PCR was performed on the TXNRD1 gene and its products were run through gel electrophoresis and purified using the QIAQuick PCR Purification Kit (Qiagen, Germantown, MD, USA). Gibson HiFi assembly (Thermo Scientific, Waltham, MA, USA) was used with the purified PCR products to give the selected TXNRD1 gene in the pET28a plasmid. The resulting plasmid was transformed into TOP10 competent *E. coli* cells and incubated overnight at 37 °C on agar plates containing 50 µg/mL of kanamycin. A colony from the plate was used to inoculate 5 mL starter cultures of LB supplemented with kanamycin and left to grow overnight at 37 °C at 220 rpm. Plasmid purification, using the QIAprep Spin Mini Prep Kit, was used to isolate the plasmid DNA. Proper plasmid construction was confirmed by sequencing from Plasmidsaurus. The plasmid was transformed into *E. coli* (DE3) BL21 for protein expression.

Expression and purification was carried out as described previously (in the supplemental materials of reference [37]). Starter cultures were prepared using colonies from the aforementioned transformation into *E. coli* (DE3) BL21, containing 10 mL of LB medium, supplemented with 50 µg/mL of kanamycin, and incubated at 37 °C under 200 rpm agitation, overnight. They were used to inoculate 1 L of expression cultures containing TB media and 50 µg/mL of kanamycin. Flasks were incubated under 200 rpm agitation at 37 °C until the OD_600_ of ~0.8 was reached, after which the temperature was dropped to 18 °C for one hour. The expression was then induced with 1 mM of isopropyl β-D-1-thiogalactopyranoside (IPTG). The cultures were incubated for approximately 18 h at 18 °C under the same agitation conditions. They were subsequently centrifuged at 4000× *g* for 1 h at 4 °C, and *E. coli* pellets were resuspended in Suspension Buffer (500 mM NaCl, 10% glycerol, 20 mM Tris pH 8.0), and frozen for future purification.

Thawed *E. coli* pellets containing the TXNRD1 protein were lysed via sonication with pulses of 15 s on and 45 s off at 80% of amplitude, for 8 min. Cells were kept in an ice bath during the sonication process. After sonication, the lysate was centrifuged at 15,000× *g* for 30 min at 4 °C. A HisTrap HP 5 mL column (Cytiva, Marlborough, MA, USA) was regenerated with 40 mL of 100 mM EDTA, 40 mL of water, 40 mL of 100 mM Ni(II) solution and washed with 30 mL of Wash Buffer (1 M NaCl, 30 mM imidazole, 20 mM Tris pH 8.0). The clarified supernatant was then applied to this column to be accessed by affinity chromatography. The column was then washed with 30 mL of Wash Buffer. His6-TXNRD1 protein was trapped in the column due to the interaction between the histidine tag and Ni(II) present in the column. The bound protein was then eluted using a linear gradient of 100% Elution Buffer (1 M NaCl, 250 mM imidazole, 20 mM Tris pH 8.0) over 40 mL, and fractions of 5 mL were collected using an ÄKTAGo FPLC system (Cytiva). Fractions were verified via a 12% SDS-PAGE gel according to purity and combined for the next purification step. Selected fractions were then concentrated in Amicon Ultra-15 30 kDa spin filters (Millipore Sigma, Burlington, MA, USA) at 4000× *g* in rounds of 10 min each, yielding ~2 mL concentrated solution. The protein was then purified via size exclusion chromatography. A pre-equilibrated HiLoad 16/60 Superdex 200 size exclusion column (Cytiva) with GF Buffer (20 mM HEPES pH 8.0, 300 mM NaCl, 10% glycerol, 20 µM PLP) was used to apply the pre-purified protein. Using the same ÄKTAGo FPLC system (Cytiva), the fraction containing TXNRD1 protein was collected, combined, and concentrated with Amicon Ultra-15 30 kDa spin filters under the same centrifugation conditions. A 12% SDS-PAGE gel was also used to check protein purity and concentration was checked via Bradford assay using Bovine Serum Albumin standards. Around ~1.5 mL of protein at 17.6 mg/mL was stored at −80 °C in aliquots of 30 µL each.

### 2.3. Cleavage of Recombinant Peptides

50 µL reaction mixtures for each peptide were generated consisting of the M^pro^ buffer, M^pro^ solution, and peptide solution. A 180 µL M^pro^ solution was prepared using 2 µL of SARS-CoV-2 M^pro^ from BPS Bioscience, reconstituted at a concentration of 1 mg/mL, and serially diluted in M^pro^ buffer (BPS Bioscience, San Diego, CA, USA) to achieve an overall concentration of 5 ng/µL. The serial dilution was performed in a 1:10, 2:20, 3:30, 16:80, and 60:180 stepwise fashion, resulting in a dilution factor of 15,000. 200 µM solutions of each peptide were prepared by reconstituting the lyophilized samples from GenScript (Piscataway, NJ, USA) in UltraPure H_2_O.

A 12-mer positive control for this assay was synthesized by GenScript to emulate the SARS-Cov-2 nsp4/nsp5 cleavage site, TSAVLQ/SGFRKM. This is the standard positive control peptide used in studies of this enzyme, as it has the highest relative catalytic efficiency of all of wild-type non-structural protein (nsp) junction sites in SARS-CoV and SARS-CoV-2 that are cleaved by M^pro^ [38,39].

Seven 50 µL samples were prepared incorporating the three solutions described above. These included two blanks, one SARS-CoV-2 M^pro^ sample, one positive control sample with M^pro^, one positive control sample without M^pro^, one proposed peptide sample with M^pro^, and one proposed peptide sample without M^pro^ as a negative control. The concentrations for M^pro^ and peptide for each sample were 15 nM and 50 µM, respectively. The composition of each sample is detailed in Table 1. Assays were incubated overnight in a PCR Thermal Cycler at 37 °C to mimic physiological conditions.

### 2.4. Cleavage of TXNRD1 Protein

Preparation of the TXNRD1 reaction mixtures differed from that of the peptide reaction mixtures only in the overall concentrations of TXNRD1 and M^pro^ in each sample. While still generating 50 µL reaction mixtures, the concentrations of TXNRD1 and SARS-CoV-2 M^pro^ were 100 µM and 0.5 µM, respectively.

### 2.5. Cleavage Analysis via UPLC-MS

The reaction mixtures were analyzed via UPLC-MS with an Acquity UPLC (Waters, Milford, MA, USA) and an Acquity UPLC BEH-C18 reverse phase column (50 mm × 2.1 mm) using 0.1% formic acid in water (A) and acetonitrile (B) for the mobile phase. The following gradient was used: 0 min, 10% B; 1.50 min 10% B; 6.50 min, 60% B; 7.00 min, 60% B; 8.00 min, 100% B; 9.00 min, 100% B; 9.10 min, 10% B; 10.00 min, 10% B. The mass spectrometric analysis was performed in a Q-Exactive Plus (Thermo Scientific, Waltham, MA, USA) in positive ion mode with a mass range of 300–2000 *m/z*. The LC-MS data was analyzed via the Qual Browser Thermo XCalibur software (v.3.0.63, Thermo Scientific). Calculated and observed masses are reported in the Results with error as Δm in PPM.

## 3. Results

### 3.1. Validation of Proposed Cleavage Sites

Peptide sequences in the 10–12-mer range incorporating the proposed cleavage sites for each subject protein were initially used to assess the validity of each site. UPLC-MS was utilized to analyze the products of each expected cleavage as well as any unexpected peptide fragments. Cleavage was deemed successful if both the abundance of intact peptide dropped and the two corresponding digested peptide fragments were detected in the peptide + protease sample, when compared with the (undigested) peptide sample.

#### 3.1.1. Assessment of Positive Control Peptide Cleavage

As expected, SARS-CoV-2 M^pro^ cleaved the positive control peptide, as evidenced by a large decrease in the abundance of the intact peptide when incubated with the protease (Figure 2A). The corresponding *m/z* values for the P and P′ fragments of the positive control (*m*/*z* = 618.3456 and 363.1911, respectively) were observed in the peptide + protease solution, but not in the peptide solution, thus not only confirming the occurrence of a cleavage, but one that occurred in the proposed position (Figure 2B,C).

#### 3.1.2. Assessment of TXNRD1 Peptide Cleavage

The 10-mer peptide, ASILQ/AGCSG was designed to represent the cleavage site within TXNRD1, which most closely resembles the nsp12/13 cleavage site (TVLQ/A). However, for reasons previously noted, the TXNRD1 active site selenocysteine in the P4′ position was replaced by a serine. Note that a 10-mer rather than a 12-mer peptide was used, because the TXNRD1 protein only extends five residues past the predicted cleavage site.

**Figure 3:**
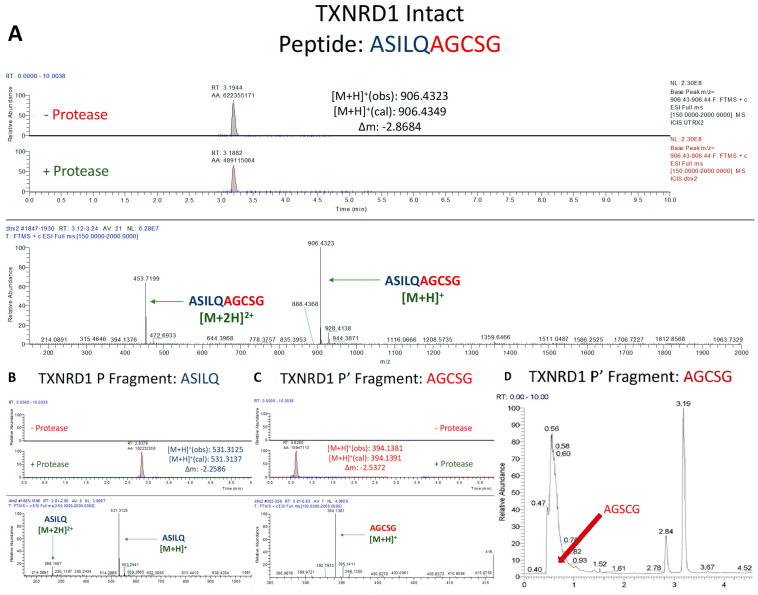
**UPLC-MS Data for TXNRD1 Peptide**. Depicted are the extracted ion chromatograms for the samples without protease and with protease (**top**) and the mass spectra (**bottom**) for (**A**) the Intact Peptide, (**B**) the P Fragment, and (**C**) the P′ Fragment. The normalization levels of the base peaks for the P and P′ fragments were 2.5 × 10^8^ and 6.15 × 10^7^, respectively. The low AGCSG peak in the mass spectrum is explained by (**D**) the large number of other compounds that have similar retention time. High resolution mass spectrometric analysis corroborates the identities of each base peak in the corresponding extracted ion chromatogram.

Incubation of the TXNRD1 derived peptide with SARS-CoV-2 M^pro^ led to a definitive cleavage (Figure 3A), albeit at a much lower efficiency than that of the positive control (Figure 2A). The less efficient processing of this peptide can be explained in three ways. First, the nsp12/13 site, to which the site in TXNRD1 is most similar, is known to be cleaved by SARS-CoV-2 M^pro^ with a low relative catalytic efficiency (8% compared to the positive control peptide from nsp4/5) [27,38,39]. Second, as observed previously [27], the core of the TXNRD1 peptide sequence (SILQA) has mismatches when compared to that of nsp12/13 (TVLQA), at the P4 and P3 positions. Although the residues at these sites are both homologous and as a pair are isosteric (SI vs. TV), a serine at P4 is not a variation that is observed in the M^pro^ consensus sequence (Figure 1B), which could also potentially reduce the cleavage efficiency. Lastly, it is possible that the substitution of the selenocysteine with serine may have also caused M^pro^ to cleave the peptide inefficiently, although this is unlikely given the relative lack of conservation of the sequence in that position, P4′ (Figure 1B). Nonetheless, cleavage of the TXNRD1 peptide was confirmed by both the prominent presence of its P and P′ fragments in the peptide + protease sample, and the absence of those fragments in the peptide only sample (Figure 3B,C).

#### 3.1.3. Assessment of GCLC Peptide Cleavage

**Figure 4:**
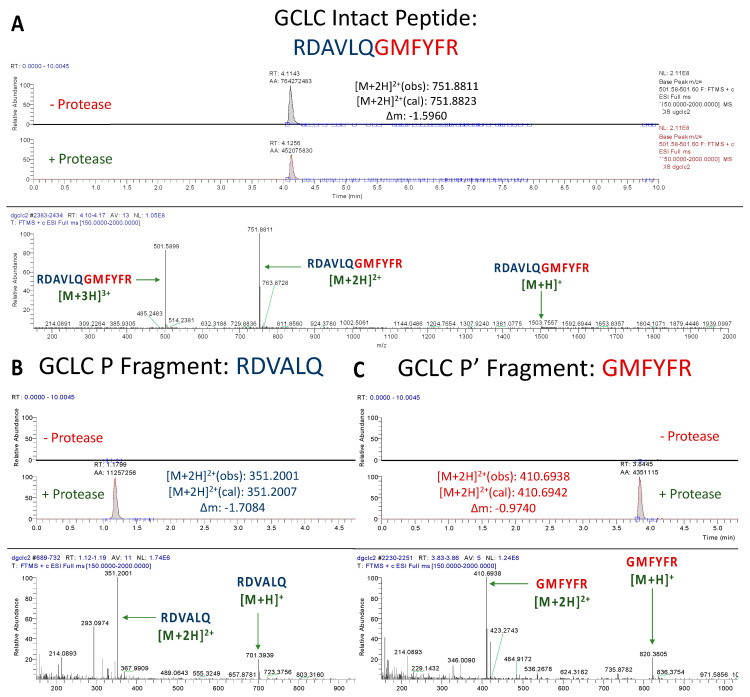
**UPLC-MS Data for GCLC Peptide.** Depicted are the extracted ion chromatograms for the samples without protease and with protease (**top**) and the mass spectra (**bottom**) for (**A**) the Intact Peptide, (**B**) the P Fragment, and (**C**) the P′ Fragment. Normalization levels of the base peaks for the P and P′ fragments were 3.20 × 10^6^ and 1.40 × 10^6^, respectively. High resolution mass spectrometric analysis corroborates the identities of each base peak in the corresponding ion chromatogram.

Incubation of the GCLC peptide, RDAVLQ/GMFYFR with SARS-CoV-2 M^pro^ led to a much greater drop in the abundance of the intact peptide than that of TXNRD1 (Figure 4A). This makes sense, as the proposed cleavage site (AVLQ/G) most closely resembles that of the well processed nsp4/5 (AVLQ/S) site. While the observed turnover appeared lower than that of the positive control (also resembling nsp4/5 cleavage site), this was most likely due to the mismatch at the P1′ position. It is known that having a serine in the P1′ position leads to the most efficient cleavage due to the stabilization offered by hydrogen bonding [40]. Ultimately, a definitive cleavage at the proposed site was established by the intense presence of the anticipated P and P′ fragments in the peptide + protease solution (Figure 4B,C).

#### 3.1.4. Assessment of SelenoP Peptide Cleavage

**Figure 5:**
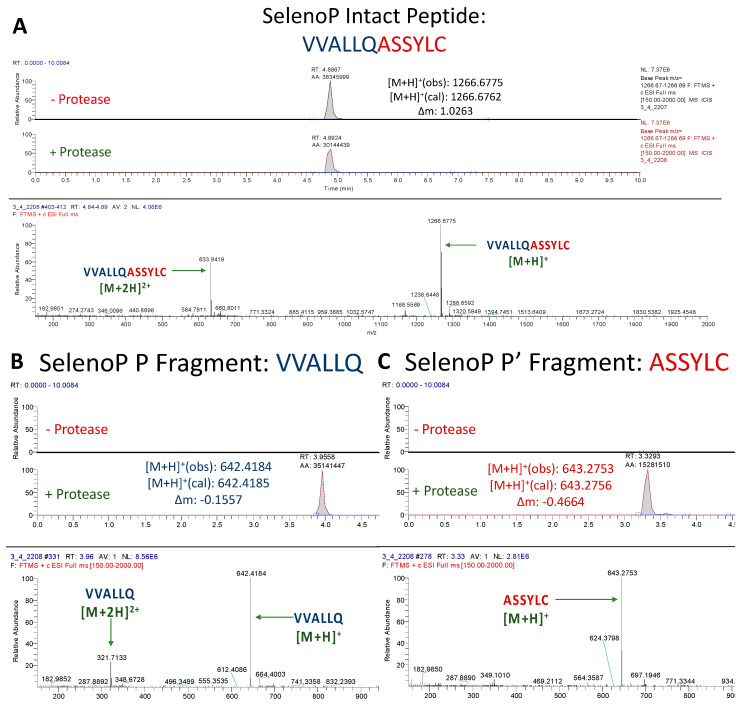
**UPLC-MS Data for SelenoP Peptide.** Depicted are the extracted ion chromatograms for the samples without protease and with protease (**top**) and the mass spectra (**bottom**) for (**A**) the Intact Peptide, (**B**) the P Fragment, and (**C**) the P′ Fragment. Normalization levels of the base peaks for the P and P′ fragments were 2.81 × 10^6^ and 8.56 × 10^6^, respectively. High resolution mass spectrometric analysis corroborates the identities of each base peak in the corresponding extracted ion chromatogram.

The SelenoP peptide (VVALLQ/ASSYLC) was cleaved at a similar level as the TXNRD1 peptide, resulting in a modest decrease in the abundance of the intact peptide upon incubation (Figure 5A). This can again be explained by both the various efficiencies by which M^pro^ cleaves its canonical cleavage sites and the mismatches in residues. The core SelenoP peptide sequence (ALLQ/A) most closely resembles that of nsp7/8 (ATLQ/A), which is cleaved by M^pro^ with a relative catalytic efficiency of 5% compared to nsp4/nsp5 [27,38,39]. Therefore, although the only mismatch between the two sequences is in the less important P3 position, the efficiency by which that viral site is cut is so low that it accounts for the slow digestion of SelenoP. However, the mismatch at the P3 position could have further decreased the efficiency by which it was cut. Nonetheless, prominent peaks were observed for the P and P′ fragments of this peptide, confirming its cleavage (Figure 5B,C).

#### 3.1.5. Assessment of SelenoF Peptide Cleavage

**Figure 6:**
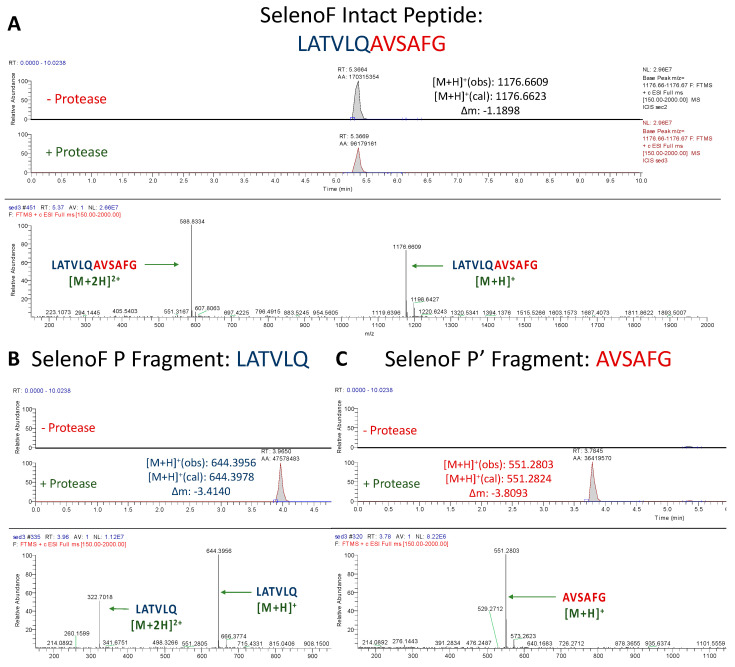
**UPLC-MS Data for SelenoF Peptide.** Depicted are the extracted ion chromatograms for the samples without protease and with protease (**top**) and the mass spectra (**bottom**) for (**A**) the Intact Peptide, (**B**) the P Fragment, and (**C**) the P′ Fragment. The base peaks for the P and P′ Fragments had normalization levels of 2.96 × 10^7^ and 1.12 × 10^7^, respectively. High resolution mass spectrometric analysis corroborates the identities of each base peak in the corresponding extracted ion chromatogram.

Similar to GCLC, the SelenoF peptide (LATVLQ/AVSAFG) exhibited a substantial drop in abundance upon incubation with M^pro^ when compared to the non-protease solution (Figure 6A). The reason for this is evident. The proposed core P4–P1′ cleavage site for SelenoF (TVLQ/A) is 100% identical to that of nsp12/13 [27]. Although nsp12/13 is cleaved by M^pro^ with only an 8% relative catalytic efficiency compared to nsp4/ nsp5 [27,38,39], the absence of mismatches allows for the SelenoF peptide to be cleaved at a higher efficiency than TXNRD1. As with the other confirmed cleavage sites, base peaks for the proposed P and P′ fragments of SelenoF were observed at high intensities, and were absent in the control samples, confirming the cleavage of yet another selenoprotein (Figure 6B,C).

#### 3.1.6. Assessment of GLRX-1 Peptide Cleavage

Unlike the results obtained for GCLC and the three selenoprotein peptides, we concluded that, despite its sequence similarities to known target sites, the proposed GLRX-1 M^pro^ target peptide does not contain a functional SARS-CoV-2 M^pro^ cleavage site. After multiple assessments, using various concentrations, no cleavage was observed. This is most likely because of a poor fit to the protease active site due to the size of the glutamine (Q) in the P1′ position, since the S1′ subunit of SARS-CoV-2 M^pro^ is quite shallow [40]. Therefore, although homologous to the asparagine (N) in the P1′ position of nsp8/9, a glutamine here most likely does not fit into the active site and was thus not cleaved.

#### 3.1.7. Assessment of GPX1 Peptide Cleavage

After multiple attempts, as for the GLRX-1 peptide, we saw no evidence of cleavage of the GPX1 10-mer peptide under the conditions used. However, there are several possible explanations for this negative result that leave open the possibility that under certain in vivo conditions, this site in GPX1 can be cleaved by Mpro. These considerations will be discussed below.

### 3.2. In Vitro Cleavage of Recombinant TXNRD1 Protein by SARS-CoV-2 M^pro^

#### Assessment of TXNRD1 Protein Cleavage

**Figure 7:**
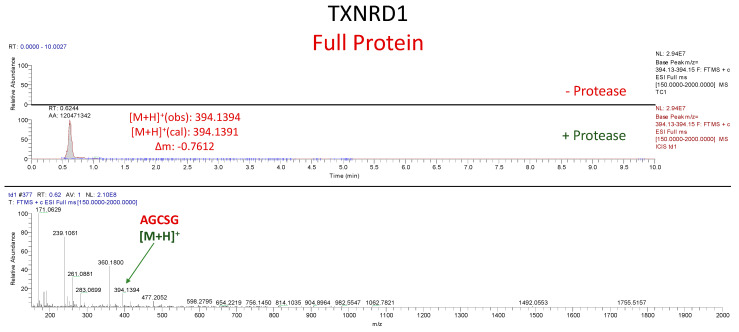
**UPLC-MS Data for TXNRD1 Protein.** Depicted are the extracted ion chromatograms for the samples without protease and with protease (**top**) and the mass spectrum for the P′ Fragment (**bottom**). High resolution mass spectrometric analysis corroborates the identities of each base peak in the corresponding extracted ion chromatogram.

To evaluate whether our results with short peptide sequences translate to SARS-CoV-2 M^pro^ mediated proteolysis of a full-length target, we heterologously expressed and purified TXNRD1 U498S from *E. coli*. As with the peptides, TXNRD1 cleavage was analyzed via UPLC-MS, but in this case only the detection of the five-residue P′ fragment (AGCSG) in the protein + protease solution and its absence in the protein only solution were necessary to establish successful cleavage. This is due to the proposed cleavage site being only five residues away from its C-terminus. Detection of this fragment was only observed in the protein + protease solution, thus confirming (consistently through multiple trials) that SARS-CoV-2 M^pro^ cleaved TXNRD1 (Figure 7).

## 4. Discussion

Our results suggest that the main protease of SARS-CoV-2 targets GCLC, TXNRD1, and two additional selenoproteins, SelenoP and SelenoF, for at least partial proteolytic degradation. The fact that we do not see 100% cleavage under the conditions used (which is true even for the positive control peptide, as seen in Figure 2A) may reflect numerous factors, including the low concentration of enzyme relative to substrates, under non-physiological conditions. In addition, isolated peptides have considerable flexibility and may not present the optimal 3D conformation that they would possess when constrained within a protein structure, and thus may be inherently less efficient at binding to the enzyme active site than in an actual protein. Furthermore, variations in the extent of cleavage within a given period of time may simply be a reflection of programmed variations in catalytic efficiency, which is characteristic of the M^pro^ enzyme with regard to its viral targets at the junctions of various nonstructural proteins, as reflected in the relative *k_cat_*/*K_m_* values for the known target sites [37,38], tabulated in Figure 2 of reference [27]. These range from the 100% normalized N-terminal site (nsp4/5) required for autocatalytic (self) cleavage of M^pro^ itself, to 41% at the M^pro^ C-terminal site (nsp5/6), to as low as 4–9% at the sites that best match the sites we have identified in TXNRD1, SelenoP, and SelenoF, which all have a consensus core sequence of LQ/A. We should also note that the TXNRD1 cleavage site is most similar to a viral site (nsp12/13) with a low 8% relative catalytic efficiency, whereas we find the GCLC peptide, which is most similar to the nsp4/5 site, to be much more completely cleaved than TXNRD1 under similar conditions. Thus, these results are consistent with the activity of M^pro^ on the native viral targets.

In regard to our negative result for the GPX1 peptide (Section 3.1.7), there are some additional considerations. Although the possibility of targeting of GPX1 by M^pro^ was the initial stimulus for this investigation [27], based on a protein-protein interaction reported by Gordon et al. [41], the expected cleavage was not observed in the 10-mer GPX1-derived peptide, from a region of GPX1 having similarities to the SARS-CoV-2 nsp13/14 cleavage site (Figure 1, and as shown more extensively in Figure 1C of reference [27]). However, this negative result is arguably inconclusive, since the conditions that may be necessary for the peptide to bind to the M^pro^ active site were absent. As previously discussed [27], glutamine (Q) appears to be the only known allowable residue in the P1 position (Figure 1B) in order for a peptide to bind and be subsequently cleaved by M^pro^. Instead, GPX1 has a selenocysteine in this position, which is significantly different from glutamine. However, as part of a series of reaction intermediates, GPX1 has been shown to form a selenylamide in this position [42], which bears a much closer resemblance to glutamine, which is a carboxamide. Therefore, further research should be done in live cells in order to allow the conditions necessary for these enzymatic reactions to occur, before GPX1 is completely ruled out as an M^pro^ target. This is further justified by previous investigations: in addition to the results of Gordon et al., which implicate GPX1 as at least a *potential* M^pro^ substrate [41], the functional consequences of expression in a transfected cell line of the homologous M^pro^ enzyme from the 2003 SARS-CoV, which include NF-κB activation, are highly consistent with the possibility of increased oxidative stress that would result from GPX1 inactivation [43].

The current findings are also consistent with the results of Wang et al., showing that SARS-CoV-2 infection causes a significant knockdown of six host selenoproteins at the mRNA level, including GPX4, TXNRD3, and four ER-resident selenoproteins, of which the SelenoF mRNA showed the most significant decrease (> 4-fold) in infected Vero cells [26]. Combined with our finding that SelenoF is also a target for virus-mediated proteolytic degradation (Section 3.1.5), one must conclude that the knockdown of this specific selenoprotein is of particular importance for some aspects of viral replication. Coronaviruses like SARS-CoV-2 are known to initiate their assembly in the ER, and ER stress is a characteristic feature of the infection at the cellular level. The potential significance of the knockdown of ER-resident selenoproteins like SelenoF in COVID-19 pathogenesis has been discussed previously [26].

Another important selenoprotein that we have shown to be a probable M^pro^ substrate is SelenoP (Section 3.1.4), which functions both as a carrier protein that is essential for the distribution of Se throughout the body, and also works enzymatically via an N-terminal redox center, whose exact roles are poorly understood [44]. It is the latter function that is likely to be disrupted by M^pro^ cleavage, given the close proximity of the cleavage site to the most N-terminal selenocysteine residue of SelenoP [27]. Nonetheless, it is of considerable interest that in COVID-19 patients, there may be a specific knockdown of SelenoP. Possible direct evidence for this effect is the observation reported by Schomburg and coworkers [10,18] that SelenoP concentrations are lower in COVID-19 cases than in uninfected controls, and decrease with increasing disease severity. In particular, in Figure 3B of reference [10] showing time resolved changes in SelenoP levels in surviving vs. deceased patients, there is a progressive decline in SelenoP in non-surviving patients. It is possible that this decrease may in part be the result of M^pro^ mediated proteolysis, which would be more pronounced at higher viral loads, i.e., in more severe cases.

In regard to the targeting of GSH biosynthesis via proteolytic degradation of GCLC (Section 3.1.3), the rate limiting enzyme for GSH biosynthesis, it must be noted that there have been a number of reports implicating GSH deficits as a major factor in COVID-19 severity and pathogenesis. COVID-19 patients have been found to manifest severe GSH deficiency, increased oxidative stress, and oxidant damage relative to uninfected controls [45]. Other authors have pointed out that low GSH levels are characteristic of many comorbid conditions associated with increased severity of COVID-19 [46], and that there are multiple molecular mechanisms by which “GSH depletion may have a fundamental role in COVID-19 pathophysiology” [47]. Quite early in the pandemic, it was presciently suggested by Polonikov that endogenous GSH deficiency was the “most likely cause of serious manifestations and death in COVID-19 patients” [48].

All of these observations become even more relevant to COVID-19 pathogenesis in light of our finding that the virus is actively targeting GSH biosynthesis for disruption. Given that GSH is an essential and ubiquitous small molecule antioxidant and free radical scavenger, with demonstrated anti-inflammatory and antiviral effects [49,50], it is clear that a significant decrease in GSH biosynthesis would have various deleterious effects consistent with COVID-19 pathologies. Direct evidence in support of our findings is the study of Bartolini et al., showing impaired metabolism and redox function of cellular GSH in SARS-CoV-2-infected Vero cells [51]. At six hours post-infection, they reported a transient increase in Nrf2 expression and protein levels of GCLC (which is upregulated by Nrf2); this effect is probably related to an initial cellular innate immune response [52]. By 24 h, Nrf2 had returned to baseline, but GCLC levels had decreased by 30% below uninfected controls (*p* < 0.05). This is consistent with the possibility of viral knockdown by proteolysis that our results imply, and would contribute directly to the observed disruption of GSH function [51].

A shared role of GSH, TXNRD1 via its dithiol substrate thioredoxin (TRX), and SelenoF via its role in protein folding quality control [53], is the reduction of disulfide bonds in proteins. This property can disrupt the structure of viral proteins such as the SARS-CoV-2 spike protein, as has been demonstrated for GSH and other thiol reductants [54], leading to decreased melting temperature of the spike protein receptor binding domain (RBD), reduced binding affinity of the RBD to its receptor ACE2, and decreased infectivity [55], probably via inhibition of fusion and viral entry [56]. These antiviral effects of GSH would be decreased by proteolytic knockdown of GCLC, favoring viral replication. Targeting of TXNRD1 and SelenoF by M^pro^ may have similar benefits for the virus, as they also, directly or indirectly (as TRX), mediate reduction of diverse disulfide substrates.

**Figure 8:**
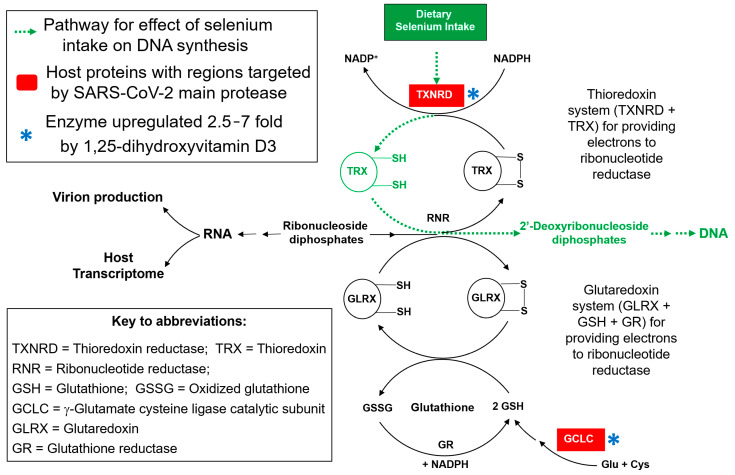
**The synthesis of DNA from RNA precursors** (ribonucleotides, as ribonucleoside diphosphates, center) depends upon their reduction by RNR to produce 2′-deoxyribonucleotides. Electrons for reduction by RNR come from either TRX or GLRX, the reduced forms of which must be constantly regenerated by one of the two essential redox cycles that sustain DNA synthesis: the TRX and GLRX systems. The former requires the selenoprotein TXNRD1, and the latter, GSH, a product of GCLC. Both TXNRD1 and GCLC are targeted by M^pro^, consistent with a viral strategy to inhibit DNA synthesis, to conserve the pool of ribonucleotides for increased virion production [33]. Significantly, in an opposing action, both of these targets are also upregulated at the mRNA level by 1,25-dihydroxyvitamin D3, which may contribute to the reported benefits of vitamin D in COVID-19 [5,6,57].

There is another biological role of GSH that is particularly significant for RNA virus replication, which could make it a primary target for disruption by SARS-CoV-2: its role as a component of one of the two essential redox systems that sustain DNA synthesis (Figure 8). DNA synthesis is inherently antithetical to the replication of RNA viruses like SARS-CoV-2, because it uses the same starting materials as those required for RNA synthesis: ribonucleotides. This is the *only* way to make DNA in all the kingdoms of life, via the enzyme ribonucleotide reductase (RNR), which utilizes several small thiol-containing proteins, TRX and glutaredoxin (GLRX), as reducing agents to convert ribonucleotides into 2′-deoxyribonucleotides.

As shown in the redox scheme of Figure 8, sustaining those reactions requires either GSH, to propel the GLRX cycle, or thioredoxin reductase (particularly the TXNRD1 isoform) to maintain the supply of reduced TRX. At least one of those two redox cycles needs to be functional to sustain the production of DNA.

Thus, it is not likely to be a coincidence that two of the enzymes that are being targeted for degradation by M^pro^ are TXNRD1 and GCLC. By suppressing the activity of *both* the TRX and GLRX redox systems, one of which can usually serve as a backup for the other, the virus could significantly slow down the conversion of ribonucleotides to 2′-deoxyribonucleotides, and thereby enhance their bioavailability for the synthesis of RNA. This is needed for viral mRNA for protein synthesis, as well as for genomic viral RNA to incorporate into new virions. In light of this mechanism, it is possible that some of the pathologies of viral infection may simply be “collateral damage” consequent to this primary goal of enhancing viral replication. Given their multiple roles in antioxidant defense, maintenance of redox homeostasis, and immune cell functioning [58,59,60,61], it is obvious that if these two essential biological reducing agents (TXNRD1 and GSH) are disrupted, the resulting increased oxidative stress and pro-inflammatory conditions, with pathological consequences, are to be expected.

One expected consequence of inhibition of DNA synthesis is decreased capacity for cells to divide, which is a characteristic of cells transfected with a SARS-CoV-2 M^pro^ expression construct: this was such a predominant effect that Resnick et al. used the decrease in cell abundance as a metric for M^pro^ activity in a drug discovery assay, with restoration of cell numbers indicative of the presence of an active M^pro^ inhibitor [62]. This effect of M^pro^ alone in the absence of virus is consistent with the hypothesis that proteolytic targeting of host proteins is effectively inhibiting DNA synthesis (Figure 8). It is also of considerable interest that both of these targets (highlighted by an asterisk in Figure 8) are significantly upregulated at the mRNA level by the active form of vitamin D, 1,25-dihydroxyvitamin D3 [63], which is known to activate various antioxidant and anti-inflammatory response genes (both directly and indirectly, via the activation of Nrf2). By tending to increase the levels of TXNRD1 and GCLC, this effect of vitamin D would act in an opposing manner to the degradative actions of the viral protease. This offset of a potentially detrimental effect of the viral infection suggests a possible preventive benefit for a higher vitamin D status, which could help to explain reports of correlations between higher blood levels of 25-hydroxyvitamin D3 or vitamin D supplementation and a reduced risk of severe outcomes in COVID-19 [5,6,57].

## 5. Conclusions

Substantial evidence has accumulated over the last four decades of a significant role for Se status in the incidence or severity of a number of viral diseases, particularly those involving RNA viruses [12,13,14,15,16,17,18,19]. SARS-CoV-2 is not only the latest of these, but also the one for which the data has perhaps been the most compelling, and which became apparent most rapidly, within a matter of months following the emergence of the virus [9]. This was probably in part because of increased vigilance and awareness on the part of investigators, who have learned from past examples—HIV/AIDS in particular, in which a role for Se is well established [64,65,66].

Significantly, based on a combination of computational and experimental lines of evidence, our group has demonstrated the possibility of virus-host RNA-RNA antisense interactions targeting host selenoprotein mRNAs, particularly isoforms of thioredoxin reductase, as a possible factor in previous observations linking Se status to viral pathogenicity [67,68,69].

The apparent antisense targeting of thioredoxin reductases by a number of RNA viruses, which is likely to interfere with protein synthesis by a variety of possible mechanisms, was the finding that first led to the hypothesis outlined above, that some RNA viruses may exploit TXNRD knockdown to inhibit the diversion of ribonucleotides for DNA synthesis, thereby enhancing viral RNA synthesis [33], via the pathway illustrated in Figure 8. The possible targeting of selenoprotein mRNAs by viral RNA antisense interactions is also supported by the finding that six selenoprotein mRNAs are significantly downregulated in SARS-CoV-2 infected cells [26]. That study also reported in vitro evidence for an antisense interaction between a region of SARS-CoV-2 RNA and the mRNA for TXNRD3, which was suppressed by 37% in infected cells. However, the most significant selenoprotein mRNA knockdown was seen for SelenoF, which our current results suggest is also targeted by SARS-CoV-2 at the protein level (Section 3.1.5). Any benefit for the virus of SelenoF knockdown is likely to be related to its roles as an ER-resident protein (and thus a potential disruptor of viral protein assembly), so there are clearly other viral strategies at play in addition to inhibition of DNA synthesis [26]. Taken together, these previous findings support the possibility that, like a number of other RNA viruses including HIV-1, Ebolavirus, and Zika virus [67,68,69], SARS-CoV-2 may be targeting certain selenoprotein mRNAs by antisense [26,33].

However, *our current findings in regard to SARS-CoV-2 are unprecedented*, because this is the first time that any virus has been shown to target selenoprotein expression *at the protein level*, by a direct proteolytic attack. The proteolytic targeting of both TXNRD1 and GCLC, and thus GSH biosynthesis, is consistent with a coordinated two-pronged attack, disrupting both redox cycles required to sustain DNA synthesis, which strongly supports the hypothesis illustrated in Figure 8. Furthermore, the potential for significant disruption of GSH synthesis by SARS-CoV-2 is highly consistent with recent clinical observations showing profound GSH deficits in patients that correlate with the severity of COVID-19 [45,46,47], as well as in infected cells [51]. The virus also benefits from GSH knockdown by inhibiting its role as a disruptor of spike protein RBD structure and binding [54,55]. Taken as a whole, this body of evidence suggests an urgent need for further investigations of GSH repletion or complementation strategies, such as supplementation with GSH precursors like N-acetyl cysteine (NAC), or the more effective combination of NAC plus glycine [45,46], γ-glutamyl cysteine (GGC, the product of GCLC), or the GSH mimic α-lipoic acid, as potentially useful symptomatic treatments for COVID-19.

Lastly, one inescapable conclusion in regard to SARS-CoV-2 is that, of any virus known to date, it is in a class by itself as far as its innovations in the perturbation of host Se and selenoprotein-based mechanisms. It certainly gives the appearance of an escalation in the arms race between viral replication and the defensive host mechanisms of which selenoproteins are an essential part.

## Figures and Tables

**Table 1:** Sample Compositions for Peptide Cleavage Assessment.

Sample	Composition
Blank 1	50 µL of buffer solution
SARS-CoV-2 M^pro^ Sample	30 µL of SARS-CoV-2 M^pro^ solution and 20 µL of buffer solution
Positive Control Sample w/o M^pro^	10 µL of the positive control solution and 40 µL of the buffer solution
Positive Control Sample w/ M^pro^	10 µL of the positive control solution, 30 µL of the SARS-CoV-2 M^pro^ solution, and 10 µL of buffer
Proposed Peptide w/o M^pro^	10 µL of the proposed peptide solution and 40 µL of the buffer solution
Proposed Peptide w/ M^pro^	10 µL of the proposed peptide solution, 30 µL of the SARS-CoV-2 M^pro^ solution, and 10 µL of buffer
Blank 2	50 µL of buffer solution

## Data Availability

All data essential for the positive conclusions drawn here are included in the article.
